# Cynophobic Fear Adaptively Extends Peri-Personal Space

**DOI:** 10.3389/fpsyt.2014.00122

**Published:** 2014-09-03

**Authors:** Marine Taffou, Isabelle Viaud-Delmon

**Affiliations:** ^1^CNRS, UMR 9912, STMS, Paris, France; ^2^IRCAM, UMR 9912, STMS, Paris, France; ^3^Sorbonne Universités, UPMC Univ Paris 06, UMR 9912, STMS, Paris, France; ^4^Inserm, U 1127, Paris, France; ^5^CNRS, UMR 7225, Paris, France; ^6^Sorbonne Universités, UPMC Univ Paris 06, UMR S 1127, Paris, France; ^7^Institut du Cerveau et de la Moelle épinière, ICM, Social and Affective Neuroscience (SAN) Laboratory, Paris, France

**Keywords:** emotion, anxiety, cynophobia, auditory–tactile integration, multisensory integration, spatial audition, 3D sound, looming sound

## Abstract

Peri-personal space (PPS) is defined as the space immediately surrounding our bodies, which is critical in the adaptation of our social behavior. As a space of interaction with the external world, PPS is involved in the control of motor action as well as in the protection of the body. The boundaries of this PPS are known to be flexible but so far, little is known about how PPS boundaries are influenced by unreasonable fear. We hypothesized that unreasonable fear extends the neural representation of the multisensory space immediately surrounding the body in the presence of a feared object, with the aim of expanding the space of protection around the body. To test this hypothesis, we explored the impact of unreasonable fear on the size of PPS in two groups of non-clinical participants: dog-fearful and non-fearful participants. The sensitivity to cynophobia was assessed with a questionnaire. We measured participants’ PPS extent in the presence of threatening (dog growling) and non-threatening (sheep bleating) auditory stimuli. The sound stimuli were processed through binaural rendering so that the virtual sound sources were looming toward participants from their rear hemi-field. We found that, when in the presence of the auditory dog stimulus, the PPS of dog-fearful participants is larger than that of non-fearful participants. Our results demonstrate that PPS size is adaptively modulated by cynophobia and suggest that anxiety tailors PPS boundaries when exposed to fear-relevant features. Anxiety, with the exception of social phobia, has rarely been studied as a disorder of social interaction. These findings could help develop new treatment strategies for anxious disorders by involving the link between space and interpersonal interaction in the approach of the disorder.

## Introduction

Peri-personal space (PPS) is defined as the space immediately surrounding our bodies (1), through which interaction with the external world occurs. PPS is opposed to the more distant, extra-personal space. Studies on both monkeys and humans have supported this distinction by showing that stimuli within PPS are represented distinctly in the brain from stimuli within extra-personal space (2). In the field of social psychology, this space near the body is referred to as “personal space” and has been described as an area with invisible boundaries that individuals actively maintain around themselves, into which the intrusion of unwanted stimulation causes discomfort (3, 4). It has been proposed that one of the roles of PPS is to implement a safety margin, which allows for the preparation and coordination of defensive behaviors against unwanted intrusions (2, 5).

Recent studies have brought evidence that the boundaries of PPS are flexible. For example, PPS can be extended through tool-use (6–8), by satisfying social interaction with others allowing integrating them to one’s PPS (9) or by depriving individuals of auditory cues from the external world (10). PPS can also be shrunk by increasing the effort needed to perform a hand movement with wrist weights (11) or by listening to positive emotion-inducing music through headphones leading to a better tolerance of others’ proximity (12).

In the present study, we investigated whether PPS size is influenced by anxiety. We hypothesized that the disproportionate experience of fear observed in some anxious disorders may be linked to the introduction of the fear-object in the boundaries of the individual’s exaggerated PPS. We explored the impact of cynophobic-based anxiety, i.e., the excessive fear of dogs on the size of PPS in two groups of non-clinical participants: dog-fearful and non-fearful participants. We recruited two groups of individuals – individuals sensitive to cynophobia [dog-fearful (DF) group] and individuals non-sensitive to cynophobia [non-fearful (NF) group] – and measured the extent of their PPS in the presence of threatening (dog growling) and non-threatening (sheep bleating) auditory stimuli looming from the rear hemi-field. Participants performed a tactile detection task with their left hand while the task-irrelevant sounds were looming toward them from the rear hemi-field. The measure of rear PPS boundaries with this audiotactile task is particularly appropriate since the auditory component of looming stimuli is especially relevant in the rear hemi-field, where the visual monitoring is not possible.

## Materials and Methods

### Participants

Participants were selected on the basis of their scores on a questionnaire exploring the fear of dogs (13). The minimal score on this dog fear questionnaire is 0, with a maximum of 42. Four hundred eighteen individuals (236 females; age: *M* = 28.87, SD = 10.44) completed this questionnaire. A mean dog fear score (*M* = 11.67, SD = 9.19) as well as a median dog fear score (Median = 8) were obtained from the questionnaire results, which served as a basis to select participants for the current experiment. Thirty healthy individuals (see details in Table 1) with normal audition and touch participated in the study. All of them were right-handed. None of them had a history of psychiatric disorders, neurological disorders or was currently undergoing medical treatment. Fifteen individuals had a low dog fear score (score <20th centile) and thus composed the NF group. The remaining 15 individuals had high dog fear scores (score >80th centile) and composed the DF group. We also used the State Trait Anxiety Inventory (STAI) (14) to measure anxiety levels. Participants completed the trait version several weeks before the experiment. All participants provided written informed consent prior to the experiment, which was approved by the Health Research Ethics Committee (CERES) of Paris Descartes University. Participants were paid 10 €/h.

**Table 1:** **Participants’ characteristics**.

Variable	All participants	NF group	DF group
Number of individuals	*N* = 30	*n*_NF_ = 15	*n*_DF_ = 15
Number of female^a^	24	10	14
Age (*M* ± SD)^a^	25.60 ± 7.73	26.93 ± 9.15	24.27 ± 6.03
95% Confidence interval	(22.71; 28.49)	(21.87; 32.00)	(20.93; 27.61)
Trait anxiety score (*M* ± SD)^b^	40.53 ± 9.79	35.53 ± 9.13	45.53 ± 7.84
95% confidence interval	(36.88; 44.19)	(30.48; 40.59)	(41.19; 49.87)
Dog fear score (*M* ± SD)^c^	15.33 ± 13.66	2.40 ± 1.64	28.27 ± 5.02
95% Confidence interval	(10.23; 20.43)	(1.49; 3.31)	(25.49; 31.05)
Range	(0.00; 36.00)	(0.00; 5.00)	(21.00; 36.00)

*^a^Both groups were similar in terms of ratio of female [χ^2^ test with Yates correction: $\chi_{\left(1\right)}^{2}=1.88$, *p* = 0.171] and age [*T*-test: *t*_(28)_ = −0.94, *p* = 0.354]*.

*^b^The trait anxiety score was significantly different between groups [*T*-test: *t*_(28)_ = 3.22, *p* = 0.003, *d* = 1.18]*.

*^c^The variance of dog fear scores was different between groups [*F*_(15,15)_ = 9.39, *p* = 0.0002], hence a non-parametric test was conducted. The dog fear score was significantly different between groups (Mann–Whitney *U*-test: *U* = 0.00, *p* < 0.001)*.

### Experimental setup and stimuli

We used a modified version of Canzoneri et al.’s audiotactile interaction task (15). Participants were blindfolded and sat on a chair with their hands palms-down on a table. Both of their hands were aligned with their mid-sagittal plane. Head movements were minimized by means of a headrest.

Auditory stimuli were presented through Sennheiser HD650 headphones. Auditory stimuli were two different (threatening and non-threatening) complex sounds (32 bits, 44100 Hz digitization). The threatening auditory stimulus was dog growling and the non-threatening one was sheep bleating. They were modified using audio editing software (Audacity software)^1^ to be continuous 3000 ms sounds and to be similar in terms of temporal dynamic and amplitude. The auditory stimuli were then processed through binaural rendering using a non-individual head related transfer functions (HRTF) of the LISTEN HRTF database^2^. With this procedure, the virtual sound source location can be manipulated by rendering accurate auditory cues such as frequency spectrum, intensity, and inter-aural differences.

The tactile stimulus was a vibratory stimulus delivered by means of small loudspeaker on the palmar surface of the left index finger of participants. A sinusoid signal was displayed for 20 ms at 250 Hz. With these parameters, the vibration of the loudspeaker was perceivable, but the sound was inaudible. A PC running Presentation^®^ software was used to control the presentation of the stimuli and to record the responses.

### Design and procedure

First, participants were invited to take part in a 20 min long diagnostic interview with a clinical psychologist based on the Mini International Neuropsychiatric Interview. This interview was conducted to ascertain that no participant met criteria for pathological anxiety disorders. Following this interview, participants were invited to evaluate the valence and arousal of the sounds used in the main experiment. Afterwards they were asked to place their left index finger on the vibrator and to press a button with their right index finger each time a tactile stimulus was detected; this constituted the main experiment. At the end of the experiment, they were asked to again evaluate the valence and arousal of the sounds.

#### Main experiment

During the main experiment, an auditory stimulus was presented for 3000 ms for each trial. The sound source approached from the rear hemi-field, either from the right (135°) or from the left hemi-space (−135°), with a spatial location varying from 520 to 20 cm from the center of the participant’s head. The auditory stimulus was preceded by 1000 ms of silence. A period of silence, with a duration varying between 2700 and 3300 ms, also occurred after the offset of the sound.

In 87.5% of the trials, a tactile stimulus was presented along with the auditory stimuli. The remaining 12.5% trials were catch trials with auditory stimulation only. Participants were instructed to ignore the auditory stimuli and to respond as quickly as possible to the tactile stimuli by pressing a button with their right index finger. They were asked to emphasize speed, but to refrain from anticipating. Reaction times (RTs) were measured.

Vibratory tactile stimuli were delivered at different delays starting from sound onset. With this procedure, the tactile stimuli were processed when the sound source was perceived at varying distances from participants’ bodies. Given that a looming auditory stimulus speeds up the processing of a tactile stimulus as long as it is perceived near the body, i.e., within PPS (15), we considered the distance at which sounds boosted tactile RTs as a proxy of PPS boundaries.

**Figure 1:**
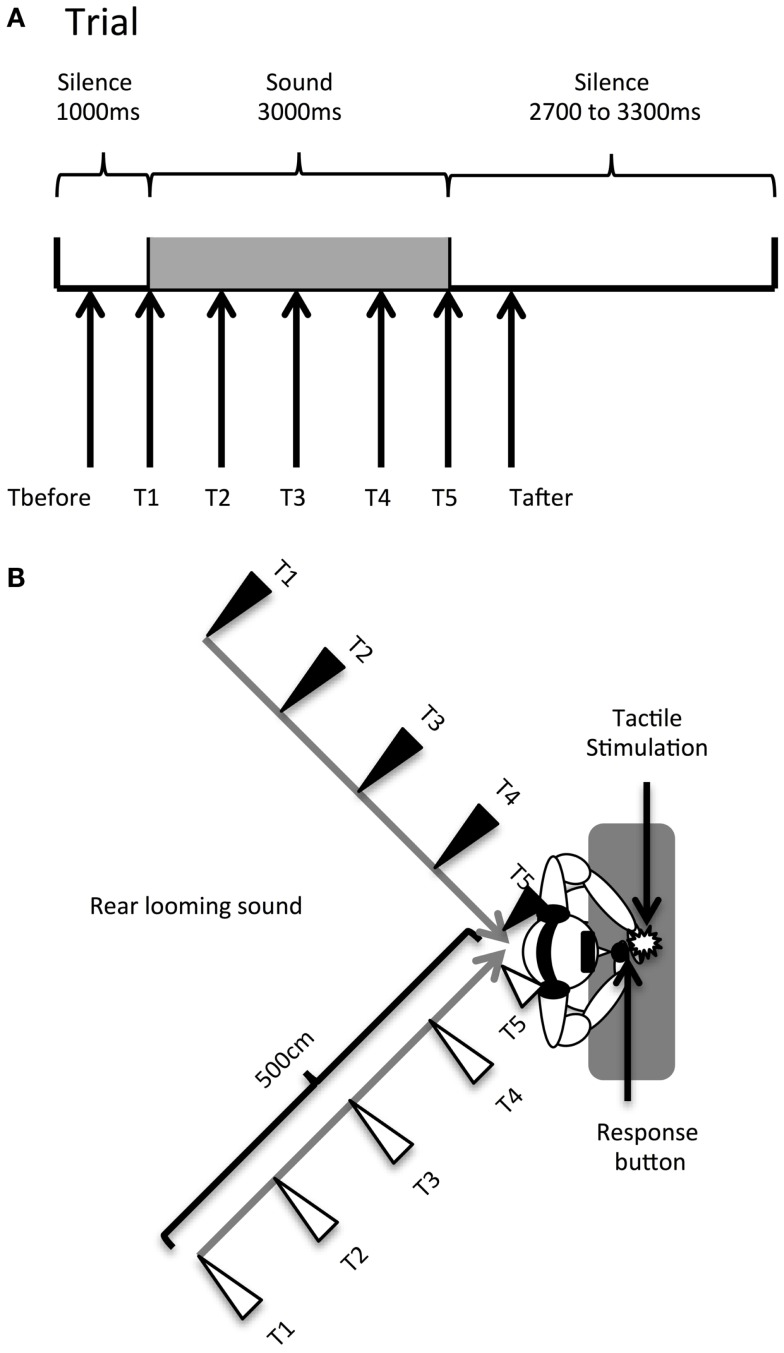
**Audiotactile test**. **(A)**. Description of a trial. **(B)** Experimental setup. Participants received a tactile stimulus at their hand while task-irrelevant sounds (threatening or non-threatening) approached them from the rear hemi-field, either in the left or the right hemi-space. When participants perceived the tactile stimulation, the looming sounds were located at different distances; this was accomplished by delivering the tactile stimulus at different temporal delays starting from sound onset (T_before_, T1, T2, T3, T4, T5, T_after_). The sound source location at each temporal delay condition is indicated by triangles (black triangles for the left hemi-space and white triangles for the right hemi-space).

Temporal delays for the tactile stimulus (see Figure 1A) were set as follows: T1 was a tactile stimulation administered simultaneously with the sound onset (corresponding to 1000 ms from the beginning of the trial); T2, at 750 ms from sound onset (at 1750 ms from trial beginning); T3, at 1500 ms from sound onset (at 2500 ms from trial beginning); T4, at 2250 ms from sound onset (at 3250 ms from trial beginning); and T5, at 3000 ms from sound onset (at 4000 ms from trial beginning). Thus, tactile stimulation occurred when the sound source was perceived at different locations with respect to the body, i.e., far from the body at low temporal delays and close to the body at high temporal delays (see Figure 1B). Moreover, in order to measure RTs in the unimodal tactile condition (without any sound), tactile stimulation was also delivered during the silent periods, preceding or following sound administration, namely at 350 ms (T_before_) and at 4650 ms (T_after_) after the beginning of the trial. The total test consisted of a random combination of eight target stimuli in each of the 28 conditions. The factors were: DELAY (seven levels: T_before_, T1, T2, T3, T4, T5, T_after_), HEMISPACE (two levels: left/right), and SOUND TYPE (two levels: threatening/non-threatening sound). There were a total of 224 trials with a tactile target, randomly intermingled with 32 catch trials. Trials were equally divided in 8 blocks of 32 trials, lasting about 4 min each. After each block, we verified that participants actually perceived the sounds as looming toward them from the rear hemi-field by directly asking them.

#### Emotional evaluation task

In order to assess any habituation phenomenon and ascertain that participants actually perceived dog growling as threatening and sheep bleating as non-threatening, participants performed a short emotional evaluation task before and after the audiotactile test. The two auditory stimuli (non-spatialized) were presented through Sennheiser HD650 headphones; each stimulus was presented only once. The order of stimuli presentation was counter-balanced between subjects. Participants had their eyes closed during the display of the sounds. After the offset of the sound, participants had to indicate the perceived valence and arousal of the sound on a 10 cm visual analogic scale (VAS).

## Results

### Emotional evaluation task

Participants’ responses on the VAS were not normally distributed for each sound stimulus. Hence, we compared the valence and arousal scores between the two sound stimuli and between groups using non-parametric tests.

**Figure 2:**
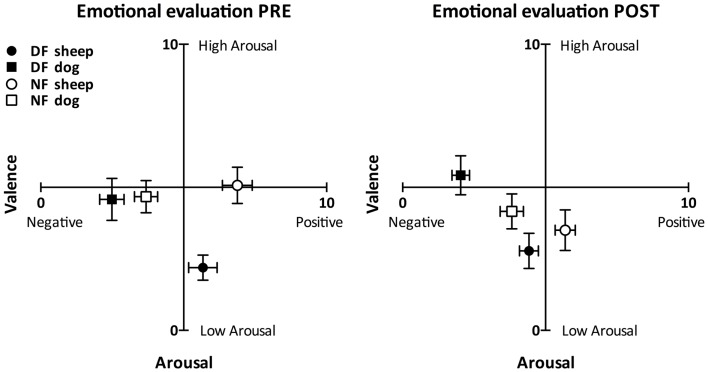
**Emotional evaluation task results**. This figure depicts the perceived arousal and valence scores (mean ± SEM) reported by the dog-fearful (in black, *n*_DF_ = 15) and non-fearful (in white, *n*_NF_ = 15) groups in response to the non-threatening (circles) and threatening (squares) sounds, in the pre- (left) and post-audiotactile task (right) emotional evaluations. The perceived valence of the dog sound was more negative than the perceived valence of the sheep sound within each group and in both emotional evaluations. Moreover, within each group, while the sheep sound was rated as positive or neutral, the dog sound was rated as negative.

As Figure 2 shows, both groups perceived the dog sound as more negatively valenced than the sheep sound in each emotional evaluation (Wilcoxon test: *T* < 9.00, *p* < 0.003 in all cases). The perceived valence of the dog sound was not different between groups before the audiotactile test (Mann–Whitney test: *U* = 69.00, *p* = 0.074) and was significantly more negative in the DF group than in the NF group after the audiotactile test (*U* = 38.50, *p* = 0.002). The perceived valence of the sheep sound tended to be more positive in the NF group than in the DF group in both emotional evaluations (*U* = 67.00, *p* > 0.058 in both cases).

The DF group perceived the dog sound as more arousing than the sheep sound (*T* < 20.00, *p* < 0.024 in both emotional evaluations), while the NF group perceived the two sounds as similarly arousing (*T* > 38.00, *p* < 0.211 in both emotional evaluations). There was no significant difference of dog sound arousal scores between the NF and the DF group (*U* > 77.00, *p* > 0.146 in both emotional evaluations). As for the sheep sound, it was perceived as more arousing by the NF group compared to the DF group before the audiotactile test (*U* = 35.00, *p* = 0.002). After the audiotactile test, there was no more significant difference of sheep sound arousal scores between the NF and the DF group (*U* = 77.00, *p* = 0.146).

The results of this control test confirmed that the dog and the sheep sounds were respectively perceived as threatening and non-threatening in both the NF and the DF groups.

### Main experiment

Two participants (one NF and one DF) were excluded from the analyses because they perceived all the stimuli as coming from the frontal hemi-field. Two participants (DF) were also excluded because their mean RTs were substantially elevated, giving us reason to suspect that they did not correctly perform the task. As the rates of false alarms and omissions were very low – 0.38 and 0.58%, respectively – participants were extremely accurate in performing the task. Consequently, the performances were only analyzed in terms of RT. One participant (DF), however, had a high rate of misses (8.48%) and was therefore excluded from the RT analyses. The analyses on the audiotactile test were conducted on the 25 remaining participants (*n*_NF_ = 14; *n*_DF_ = 11). RTs non-precise measures due to interruptions from operating systems or device drivers were trimmed from the analyses. Mean RTs to tactile targets were calculated for each DELAY level and separately for each participant. RTs exceeding more than two standard deviations from the mean RT were considered outliers and also trimmed from the analyses (4.54% of the trials).

Mean RTs to tactile target were calculated for each of the 28 conditions (2 SOUND TYPE*2 HEMISPACE*7 DELAY). We first conducted an ANOVA on the mean RTs, with the between subject factor GROUP (NF/DF) and the within subject factors SOUND TYPE (threatening/non-threatening stimulus), HEMISPACE (left/right) and DELAY (T_before_, T1, T2, T3, T4, T5, T_after_). The global effect of DELAY was significant [*F*_(6,138)_ = 31.42, *p* < 0.001, $\eta_{p}^{2}=0.577$] suggesting that RTs were influenced by the time of tactile stimulation delivery. RTs in the unimodal condition T_before_ (391.69 ± 49.23 ms) were significantly slower than RTs in the bimodal conditions T1, T2, T3, T4, and T5 (*post hoc* Newman–Keuls’ test: *p* < 0.001 in all cases). RTs in the unimodal condition T_after_ (353.92 ± 33.77 ms) were significantly faster than RTs at T_before_ (*post hoc* Newman–Keuls’ test: *p* < 0.001). Given that RTs at T_after_ were significantly slower than RTs at T5 (*post hoc* Newman–Keuls’ test: *p* < 0.001), we can exclude the possibility that participants were faster at late delays because of the increasing probability of receiving a tactile stimulation along trials. The difference in tactile RTs between T_before_ and T_after_ can be explained by the semantic content of the looming sounds, which places an animal in the environment; at T_after_, participants potentially considered the animal as close to them but silent.

We then conducted an ANOVA on the mean RTs measured in the bimodal trials only, with the between subject factor GROUP (NF/DF) and the within subject factors SOUND TYPE (threatening/non-threatening stimulus), HEMISPACE (left/right) and DELAY (T1, T2, T3, T4, T5). The global effect of DELAY was significant [*F*_(4,92)_ = 18.24, *p* < 0.001, $\eta_{p}^{2}=0.442$]. The three-way interaction GROUP*SOUND TYPE*DELAY was also significant [*F*_(4,92)_ = 4.853, *p* = 0.001, $\eta_{p}^{2}=0.174$] suggesting that RTs were differently modulated in the NF and the DF group depending on the perceived position of sound in space and as a function of whether the auditory stimulus was threatening or not. In the threatening condition, DF group’s RTs were significantly faster when the tactile stimulus occurred at T2, T3, T4, and T5 compared to when the tactile stimulus occurred at T1 (*post hoc* Newman–Keuls’ test: *p* < 0.001 in all cases). Contrastingly, in the non-threatening condition, DF group’s RTs were faster when the tactile stimulus occurred at T4 and T5 compared to when it occurred at T1, T2, and T3 (*post hoc* Newman–Keuls’ test: *p* < 0.05 in all cases). RTs at T2 were faster in the threatening condition compared to the non-threatening condition (*post hoc* Newman–Keuls’ test: *p* = 0.038). RTs were not different between the threatening and non-threatening condition for the longest delays, i.e., closest distances (T3, T4, and T5) or for the smallest delay T1, i.e., the greater distance (*post hoc* Newman–Keuls’ test: *p* > 0.217 in all cases). These results suggest that, in the DF group, the threatening sound began to affect tactile RTs at further distances compared to the non-threatening sound. In both the threatening and non-threatening condition, the NF group’s RTs were significantly faster when the tactile stimulus occurred at T5 compared to when the tactile stimulus occurred at T1, T2, T3, and T4 (*post hoc* Newman–Keuls’ test: *p* < 0.002 in all cases), suggesting that the distance at which the sound began to affect tactile RTs was similar in both the threatening and the non-threatening conditions.

**Figure 3:**
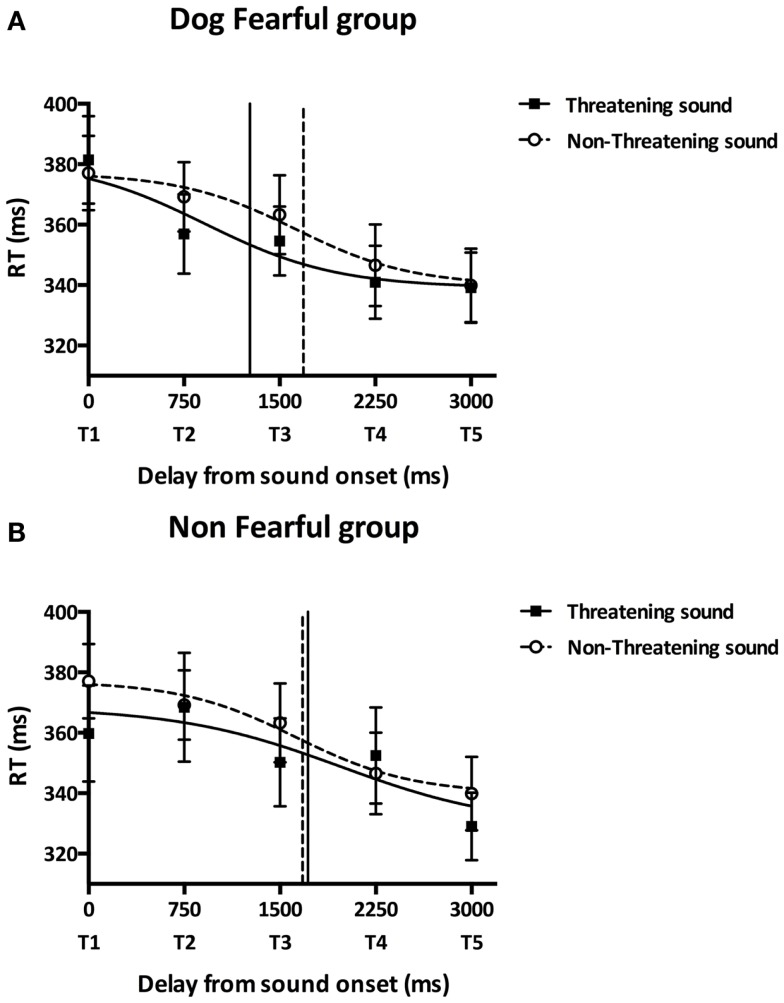
**Main experiment results**. Participants performed the audiotactile task by responding to a tactile stimulation while a task-irrelevant threatening (dog growling) or non-threatening (sheep bleating) sound was looming toward them. This figure reports the mean tactile reactions times (±SEM) for the dog-fearful (top graph) and non-fearful group (bottom graph) in the threatening (black square) or non-threatening (white circles) sound conditions as a function of the delay of tactile stimulation delivery from sound onset. Reaction times were fitted with a sigmoid function. The inflection point abscissa of the sigmoid curves was computed as a measure of the temporal delay, i.e., the distance, at which sound starts affecting tactile RTs and was analyzed in order to quantify PPS boundaries. **(A)** Dog-fearful group results. The abscissa of the curve’s inflection point was lower in the threatening sound condition (1266.81 ± 287.57 ms, black vertical line) compared to the non-threatening sound condition (1685.49 ± 548.41 ms, dashed vertical line) meaning that PPS boundaries were farther from participants in the presence of the dog sound than in the presence of the sheep sound. **(B)** Non-fearful group results. The abscissa of the curve’s inflection point did not significantly differ between the threatening (1717.70 ± 413.23 ms, black vertical line) and the non-threatening (1675.15 ± 596.56 ms, dashed vertical line) sound conditions suggesting that participants’ PPS size was similar in the presence of the dog and the sheep sounds. While the dog-fearful group’s PPS was larger than the non-fearful group’s PPS in the presence of the dog sound, there was no significant difference in PPS size between groups in the presence of the sheep sound.

In order to further investigate the influence of the different sounds on tactile RTs, we fitted participants’ mean tactile RTs at the five delays with a sigmoid function using the same procedure as Canzoneri et al. The sigmoid function was described by the following equation: $y\left(x\right)=\frac{y_{\min}+y_{\max}\times e^{\left(x-x_{i}∕b\right)}}{1+e^{\left(x-x_{i}∕b\right)}}$ where *x* represents the independent variable (i.e., the delay of tactile stimulation from sound onset in ms), *y* the dependent variable (i.e., tactile RT), *y*_min_ and *y*_max_ the lower and upper plateau of the sigmoid, *x_i_* the value of the abscissa at the inflection point of the sigmoidal curve (i.e., the value of *x* at which $y=\frac{y_{\min}+y_{\max}}{2}$) and *b* is the slope at the inflection point. We estimated the parameters *x_i_* and *b* for each participant’s in each sound condition (threatening/non-threatening) and assigned *a priori y*_min_ and *y*_max_ to the minimum and maximum values of each data set. The sigmoid function better described participants’ data than a linear function [*y*(*x*) = *y*_0_ × *x* + *a*, where *y*_0_ is the intercept at *x* = 0 and *a* is the slope) as indicated by the result of the comparison of the root mean square errors (RMSE_sigmoid_ = 7.80 ms, RMSE_linear_ = 8.69 ms, Wilcoxon test: *T* = 149.00, *p* = 0.001). The parameter *x_i_* was computed as a measure of the temporal delay, i.e., the distance, at which sound starts affecting tactile RTs and was analyzed in order to quantify PPS boundaries. As Figure 3A shows, DF group’s *x_i_* was lower in the threatening compared to the non-threatening condition [*t*_(8)_ = −1.89, *p* = 0.030, one-tailed, two participants were excluded due to bad fitting] suggesting that the boundaries of DF group’s PPS in the threatening condition are farther from the participants than in the non-threatening condition. As Figure 3B shows, NF group’s *x_i_* did not significantly differ between sound conditions [*t*_(9)_ = 0.19, *p* = 0.851, two-tailed, four participants were excluded due to bad fitting] suggesting that the NF group’s PPS size was similar in the threatening and in the non-threatening conditions. While the DF group’s PPS was larger than the NF group’s PPS in the threatening condition [*t*_(17)_ = −2.73, *p* = 0.007, one-tailed], there was no significant difference in PPS size between groups in the non-threatening condition [*t*_(17)_ = 0.04, *p* = 0.485, one-tailed]. Participants’ difference between *x_i_* in the non-threatening condition and *x_i_* in the threatening condition, i.e., the extension of PPS boundaries, was not significantly correlated with trait anxiety scores (*r* = 0.318, *p* = 0.184).

## Discussion

Approaching unpleasant sounds trigger a particularly intense emotional response suggesting an activation of defensive responses (16). Previous results demonstrated that at distances wherein individuals non-sensitive to cynophobia still feel comfortable, a virtual visual looming dog triggers high discomfort for individuals sensitive to cynophobia (17). This variance in distance, together with PPS’s proposed role of implementing a safety margin around the body, leads us to hypothesize that fear-object looming toward the body will expand PPS boundaries.

Consistently, our results suggest that looming feared elements extend PPS; the space that individuals consider as belonging to themselves enlarges when they perceive a feared object. This result seems consistent with previous results demonstrating that individuals underestimate the time at which a visual looming stimulus will collide with them when the stimulus is threatening (snakes, spiders, angry faces) compared to when it is non-threatening (butterflies, rabbits, neutral faces) (18, 19). Vagnoni et al. also show that this underestimation of time-to-collision is bigger for individuals who are fearful of the threatening stimulus; the size of the underestimation is linked to individuals’ level of snakes- and spider-related anxiety. If PPS is extended, the distance between the feared object and PPS boundaries is smaller. Consequently, the encounter with PPS occurs sooner. Thus, the fact that an approaching feared stimulus is perceived as colliding sooner seems coherent with the PPS boundaries being farther.

Peri-personal space has also been shown as being extended after a satisfying social interaction (9). In our experiment, the expansion of PPS seems to aim at keeping unwanted and potentially harmful stimuli far from the body (i.e., outside PPS) and at allowing additional time for triggering defensive behaviors. In Teneggi et al. study, individuals’ PPS boundaries did not enlarge in order to keep the other individual outside of PPS but rather to integrate them within it. In this case, the expansion of PPS would be linked to the implementation of approach behaviors.

Although PPS seems to be linked to emotional processes (20) and is thought to have a protective function, little is known on how PPS boundaries are influenced by anxiety. It has been shown that sensitivity to claustrophobic fear is related to larger PPS size as measured by a line bisection task (21). In their study, they observed a positive correlation between PPS size and the level of this space-related anxiety that is claustrophobic fear. This link was not observed with PPS size as measured by the hand-blink reflex defensive response (22). They instead observed a link between the size of PPS and trait anxiety. In contrast, results collected during a stop-approach task did not support a modulation of PPS size by anxiety (23). Our findings suggest that anxiety selectively influences PPS: sensitivity to cynophobia expands PPS boundaries when there is a dog stimulus in the environment. The diversity of results is potentially explained by the variety of experimental settings, which deliver different amount of fear-relevant features.

Though we studied a non-clinical sample, this situation-dependent effect of dog fear suggests that, at least in cynophobia, selective distortion of PPS is involved. Intrusion in PPS triggers high discomfort and regulative behaviors such as flight (5). When not constrained by the physical environment, individuals typically prevent undesired components of the environment from entering their PPS by adjusting their distance from them. Over-projecting PPS could allow more time to prepare defensive or avoidant behaviors in case of attack. The expansion of PPS in the presence of feared elements fits with the proposed protective function of PPS, i.e., assuring a margin of safety around the body (2, 5). What is perceived to be a disproportionate reaction from cynophobic individuals in the presence of dogs may be partially attributed to a normal reaction to the intrusion of an undesirable stimulus in an enlarged PPS.

Clinical psychology has implicitly used the notion of the influence of anxiety on PPS with the widely used Behavioral Assessment Test (BAT). This test is used to assess the level of fear of the patient in relation to a phobic object that is coming closer to him/her. When comparing the distance between the individual and the feared object at the beginning of therapy to the distance at the end of the therapy, the BAT serves as a measure of success [e.g., Ref. (24)]. A positive treatment outcome, as revealed by the BAT, probably reflects a change in the boundaries of PPS. The acceptable distance with the feared object is therefore a critical criterion in the assessment of severity of phobias. Our results suggest that PPS distortion could play a role in several phobias and that shrinking the oversized PPS could be a treatment strategy when facing fear-relevant situations.

Because anxiety regulation is shaped by the social context, we think it is important to take social distances into account when appraising anxiety mechanisms. Space is not a unitary construct in the brain and its neural representation is parceled across different compartments according to the behavioral interactions we have with them (25). Interactions between self and others can spread across the different compartments of space. It has already been suggested that space perception and representation might be distorted by anxiety [see Ref. (26) for a review]. While it is mainly the influence of anxiety on extra-personal space perception that has been studied [e.g., Ref. (27, 28)], it seems that PPS is another compartment of space that is distorted by anxiety.

## Conflict of Interest Statement

The authors declare that the research was conducted in the absence of any commercial or financial relationships that could be construed as a potential conflict of interest.

## Footnotes

^1^http://audacity.sourceforge.net/

^2^http://recherche.ircam.fr/equipes/salles/listen/
